# Supplementary material for: Enhancing and inhibitory motifs regulate CD4 activity
Source: eLife. 2022 Jul 21;11:e79508. doi: 10.7554/eLife.79508 (PMC9333989; doi:10.7554/eLife.79508)
Supplement: Supplementary file 2. [file elife-79508-supp2.docx]

**Constructs**

The following sequence for 5cc7α was subcloned into the pZ4 zeocin-resistance MSCV vector (MCS-IRES-Zeo) resistance through 5’XhoI and 3’EcoRI:

cctcgagcgccaccatggagaggaacctgggagctgtgctggggattctgtgggtgcagatttgctgggtgagaggagatcaggtggagcagagtccttcagccctgagcctccacgagggaaccggttctgctctgagatgcaattttactaccaccatgagggctgtgcagtggttccgaaagaattccaggggcagcctcatcaatctgttctacttggcttcaggaacaaaggagaatgggaggctaaagtcagcatttgattctaaggagcgctacagcaccctgcacatcagggatgcccagctggaggactcaggcacttacttctgtgctgctgaggcttccaataccaacaaagtcgtctttggaacagggaccagattacaagtattaccaaacatccagaacccagaacctgctgtgtaccagttaaaagatcctcggtctcaggacagcaccctctgcctgttcaccgactttgactcccaaatcaatgtgccgaaaaccatggaatctggaacgttcatcactgacaaaactgtgctggacatgaaagctatggattccaagagcaatggggccattgcctggagcaaccagacaagcttcacctgccaagatatcttcaaagagaccaacgccacctaccccagttcagacgttccctgtgatgccacgttgaccgagaaaagctttgaaacagatatgaacctaaactttcaaaacctgtcagttatgggactccgaatcctcctgctgaaagtagcgggatttaacctgctcatgacgctgaggctgtggtccagtgcggccgcatgataaagatctggatcctgactgagtgagaattc

The following sequence for 5cc7βG was subcloned into the pP2 puromycin-resistance MSCV vector (MCS-IRES-puro) resistance through 5’XhoI and 3’BamHI:

actcgagcgccaccatggctacaaggctcctctgttacacagtactttgtctcctgggtgcaagaattttgaattcaaaagtcattcagactccaagatatctggtgaaagggcaaggacaaaaagcaaagatgaggtgtatccctgaaaagggacatccagttgtattctggtatcaacaaaataagaacaatgagtttaaatttttgattaactttcagaatcaagaagttcttcagcaaatagacatgactgaaaaacgattctctgctgagtgtccttcaaactcaccttgcagcctagaaattcagtcctctgaggcaggagactcagcactgtacctctgtgccagcagtctgaacaatgcaaactccgactacaccttcggctcagggaccaggcttttggtaatagaggatctgagaaatgtgactccacccaaggtctccttgtttgagccatcaaaagcagagattgcaaacaaacaaaaggctaccctcgtgtgcttggccaggggcttcttccctgaccacgtggagctgagctggtgggtgaatggcaaggaggtccacagtggggtcagcacggaccctcaggcctacaaggagagcaattatagccactgcctgagcagccgcctgagggtctctgctaccttctggcacaatcctcgcaaccacttccgctgccaagtgcagttccatgggctttcagaggaggacaagtggccagagggctcacccaaacctgtcacacagaacatcagtgcagaggcctggggccgagcagactgtgggattacctcagcatcctatcaacaaggggtcttgtctgccaccatcctctatgagatcctgctagggaaagccaccctgtatgctgtgcttgtcagtacactggtggtgatggctatggtcaaaagaaagaattccgcggccgcaggtggaggcggatcaggtggcggtggaagtggaggtggtggatctatggtgagcaagggcgaggagctgttcaccggggtggtgcccatcctggtcgagctggacggcgacgtaaacggccacaagttcagcgtgtccggcgagggcgagggcgatgccacctacggcaagctgaccctgaagttcatctgcaccaccggcaagctgcccgtgccctggcccaccctcgtgaccaccctgacctacggcgtgcagtgcttcagccgctaccccgaccacatgaagcagcacgacttcttcaagtccgccatgcccgaaggctacgtccaggagcgcaccatcttcttcaaggacgacggcaactacaagacccgcgccgaggtgaagttcgagggcgacaccctggtgaaccgcatcgagctgaagggcatcgacttcaaggaggacggcaacatcctggggcacaagctggagtacaactacaacagccacaacgtctatatcatggccgacaagcagaagaacggcatcaaggtgaacttcaagatccgccacaacatcgaggacggcagcgtgcagctcgccgaccactaccagcagaacacccccatcggcgacggccccgtgctgctgcccgacaaccactacctgagcacccagtccaagctgagcaaagaccccaacgagaagcgcgatcacatggtcctgctggagttcgtgaccgccgccgggatcactctcggcatggacgagctgtacaagtaatgaggatcctga

Full-length CD3δ, ε, γ, and ζ were encoded on a poly-cistronic construct as previously described (Holst et al., 2008; Kuhns and Davis, 2007)

The following CD4 constructs WT, TMD, Palm, Clasp, TP, TPC, pSS, LL+pSS, and CD4-T1 were cloned by conventional molecular biology techniques, including PCR-based mutagenesis where needed, into pUC18 via 5’EcoRI and 3’HindIII. After sequence verification they were subcloned into pP2 puromycin-resistance MSCV vector (MCS-IRES-puro) vectors via 5’XhoI and 3’BglII, midi-prepped (Qiagen), and sequence verified. The sequences for these constructs are as follows. Mutated codons are shown in bold uppercase letters, while the motif under interrogation is shown in bold and underlined (any non-mutated codons within the motif are bold lowercase letters and underlined):

**WT:**

acggaattccgctcgagcgccaccatggtgcgagccatctctcttaggcgcttgctgctgctgctgctgcagctgtcacaactcctagctgtcactcaagggaagacgctggtgctggggaaggaaggggaatcagcagaactgccctgcgagagttcccagaagaagatcacagtcttcacctggaagttctctgaccagaggaagattctggggcagcatggcaaaggtgtattaattagaggaggttcgccttcgcagtttgatcgttttgattccaaaaaaggggcatgggagaaaggatcgtttcctctcatcatcaataaacttaagatggaagactctcagacttatatctgtgagctggagaacaggaaagaggaggtggagttgtgggtgttcaaagtgaccttcagtccgggtaccagcctgttgcaagggcagagcctgaccctgaccttggatagcaactctaaggtctctaaccccttgacagagtgcaaacacaaaaagggtaaagttgtcagtggttccaaagttctctccatgtccaacctaagggttcaggacagcgacttctggaactgcaccgtgaccctggaccagaaaaagaactggttcggcatgacactctcagtgctgggttttcagagcacagctatcacggcctataagagtgagggagagtcagcggagttctccttcccactcaactttgcagaggaaaacgggtggggagagctgatgtggaaggcagagaaggattctttcttccagccctggatctccttctccataaagaacaaagaggtgtccgtacaaaagtccaccaaagacctcaagctccagctgaaggaaacgctcccactcaccctcaagataccccaggtctcgcttcagtttgctggttctggcaacctgactctgactctggacaaagggacactgcatcaggaagtgaacctggtggtgatgaaagtggctcagctcaacaatactttgacctgtgaggtgatgggacctacctctcccaagatgagactgaccctgaagcaggagaaccaggaggccagggtctctgaggagcagaaagtagttcaagtggtggcccctgagacagggctgtggcagtgtctactgagtgaaggtgataaggtcaagatggactccaggatccaggttttatccagaggggtgaaccagacagtgttcctggcttgcgtgctgggtggctccttcggctttctgggtttccttgggctctgcatcctctgctgtgtcaggtgccggcaccaacagcgccaggcagcacgaatgtctcagatcaagaggctcctcagtgagaagaagacctgccagtgcccccaccggatgcagaagagccataatctcatctaatgaagatcttgagtcgacaagcttgctag

**TMD:**

acggaattccgctcgagcgccaccatggtgcgagccatctctcttaggcgcttgctgctgctgctgctgcagctgtcacaactcctagctgtcactcaagggaagacgctggtgctggggaaggaaggggaatcagcagaactgccctgcgagagttcccagaagaagatcacagtcttcacctggaagttctctgaccagaggaagattctggggcagcatggcaaaggtgtattaattagaggaggttcgccttcgcagtttgatcgttttgattccaaaaaaggggcatgggagaaaggatcgtttcctctcatcatcaataaacttaagatggaagactctcagacttatatctgtgagctggagaacaggaaagaggaggtggagttgtgggtgttcaaagtgaccttcagtccgggtaccagcctgttgcaagggcagagcctgaccctgaccttggatagcaactctaaggtctctaaccccttgacagagtgcaaacacaaaaagggtaaagttgtcagtggttccaaagttctctccatgtccaacctaagggttcaggacagcgacttctggaactgcaccgtgaccctggaccagaaaaagaactggttcggcatgacactctcagtgctgggttttcagagcacagctatcacggcctataagagtgagggagagtcagcggagttctccttcccactcaactttgcagaggaaaacgggtggggagagctgatgtggaaggcagagaaggattctttcttccagccctggatctccttctccataaagaacaaagaggtgtccgtacaaaagtccaccaaagacctcaagctccagctgaaggaaacgctcccactcaccctcaagataccccaggtctcgcttcagtttgctggttctggcaacctgactctgactctggacaaagggacactgcatcaggaagtgaacctggtggtgatgaaagtggctcagctcaacaatactttgacctgtgaggtgatgggacctacctctcccaagatgagactgaccctgaagcaggagaaccaggaggccagggtctctgaggagcagaaagtagttcaagtggtggcccctgagacagggctgtggcagtgtctactgagtgaaggtgataaggtcaagatggactccaggatccaggttttatccagaggggtgaaccagacagtgttcctggcttgcgtgctgggt**GTGtccttcCTC**tttctgggtttccttgggctctgcatcctctgctgtgtcaggtgccggcaccaacagcgccaggcagcacgaatgtctcagatcaagaggctcctcagtgagaagaagacctgccagtgcccccaccggatgcagaagagccataatctcatctaatgaagatcttgagtcgacaagcttgctag

**Palm:**

acggaattccgctcgagcgccaccatggtgcgagccatctctcttaggcgcttgctgctgctgctgctgcagctgtcacaactcctagctgtcactcaagggaagacgctggtgctggggaaggaaggggaatcagcagaactgccctgcgagagttcccagaagaagatcacagtcttcacctggaagttctctgaccagaggaagattctggggcagcatggcaaaggtgtattaattagaggaggttcgccttcgcagtttgatcgttttgattccaaaaaaggggcatgggagaaaggatcgtttcctctcatcatcaataaacttaagatggaagactctcagacttatatctgtgagctggagaacaggaaagaggaggtggagttgtgggtgttcaaagtgaccttcagtccgggtaccagcctgttgcaagggcagagcctgaccctgaccttggatagcaactctaaggtctctaaccccttgacagagtgcaaacacaaaaagggtaaagttgtcagtggttccaaagttctctccatgtccaacctaagggttcaggacagcgacttctggaactgcaccgtgaccctggaccagaaaaagaactggttcggcatgacactctcagtgctgggttttcagagcacagctatcacggcctataagagtgagggagagtcagcggagttctccttcccactcaactttgcagaggaaaacgggtggggagagctgatgtggaaggcagagaaggattctttcttccagccctggatctccttctccataaagaacaaagaggtgtccgtacaaaagtccaccaaagacctcaagctccagctgaaggaaacgctcccactcaccctcaagataccccaggtctcgcttcagtttgctggttctggcaacctgactctgactctggacaaagggacactgcatcaggaagtgaacctggtggtgatgaaagtggctcagctcaacaatactttgacctgtgaggtgatgggacctacctctcccaagatgagactgaccctgaagcaggagaaccaggaggccagggtctctgaggagcagaaagtagttcaagtggtggcccctgagacagggctgtggcagtgtctactgagtgaaggtgataaggtcaagatggactccaggatccaggttttatccagaggggtgaaccagacagtgttcctggcttgcgtgctgggtggctccttcggctttctgggtttccttgggctctgcatcctctgc**TCTgtcaggTCC**cggcaccaacagcgccaggcagcacgaatgtctcagatcaagaggctcctcagtgagaagaagacctgccagtgcccccaccggatgcagaagagccataatctcatctaatgaagatcttgagtcgacaagcttgctag

**Clasp:**

acggaattccgctcgagcgccaccatggtgcgagccatctctcttaggcgcttgctgctgctgctgctgcagctgtcacaactcctagctgtcactcaagggaagacgctggtgctggggaaggaaggggaatcagcagaactgccctgcgagagttcccagaagaagatcacagtcttcacctggaagttctctgaccagaggaagattctggggcagcatggcaaaggtgtattaattagaggaggttcgccttcgcagtttgatcgttttgattccaaaaaaggggcatgggagaaaggatcgtttcctctcatcatcaataaacttaagatggaagactctcagacttatatctgtgagctggagaacaggaaagaggaggtggagttgtgggtgttcaaagtgaccttcagtccgggtaccagcctgttgcaagggcagagcctgaccctgaccttggatagcaactctaaggtctctaaccccttgacagagtgcaaacacaaaaagggtaaagttgtcagtggttccaaagttctctccatgtccaacctaagggttcaggacagcgacttctggaactgcaccgtgaccctggaccagaaaaagaactggttcggcatgacactctcagtgctgggttttcagagcacagctatcacggcctataagagtgagggagagtcagcggagttctccttcccactcaactttgcagaggaaaacgggtggggagagctgatgtggaaggcagagaaggattctttcttccagccctggatctccttctccataaagaacaaagaggtgtccgtacaaaagtccaccaaagacctcaagctccagctgaaggaaacgctcccactcaccctcaagataccccaggtctcgcttcagtttgctggttctggcaacctgactctgactctggacaaagggacactgcatcaggaagtgaacctggtggtgatgaaagtggctcagctcaacaatactttgacctgtgaggtgatgggacctacctctcccaagatgagactgaccctgaagcaggagaaccaggaggccagggtctctgaggagcagaaagtagttcaagtggtggcccctgagacagggctgtggcagtgtctactgagtgaaggtgataaggtcaagatggactccaggatccaggttttatccagaggggtgaaccagacagtgttcctggcttgcgtgctgggtggctccttcggctttctgggtttccttgggctctgcatcctctgctgtgtcaggtgccggcaccaacagcgccaggcagcacgaatgtctcagatcaagaggctcctcagtgagaagaagacc**TCCcagTCC**ccccaccggatgcagaagagccataatctcatctaatgaagatcttgagtcgacaagcttgctag

**TP**:

acggaattccgctcgagcgccaccatggtgcgagccatctctcttaggcgcttgctgctgctgctgctgcagctgtcacaactcctagctgtcactcaagggaagacgctggtgctggggaaggaaggggaatcagcagaactgccctgcgagagttcccagaagaagatcacagtcttcacctggaagttctctgaccagaggaagattctggggcagcatggcaaaggtgtattaattagaggaggttcgccttcgcagtttgatcgttttgattccaaaaaaggggcatgggagaaaggatcgtttcctctcatcatcaataaacttaagatggaagactctcagacttatatctgtgagctggagaacaggaaagaggaggtggagttgtgggtgttcaaagtgaccttcagtccgggtaccagcctgttgcaagggcagagcctgaccctgaccttggatagcaactctaaggtctctaaccccttgacagagtgcaaacacaaaaagggtaaagttgtcagtggttccaaagttctctccatgtccaacctaagggttcaggacagcgacttctggaactgcaccgtgaccctggaccagaaaaagaactggttcggcatgacactctcagtgctgggttttcagagcacagctatcacggcctataagagtgagggagagtcagcggagttctccttcccactcaactttgcagaggaaaacgggtggggagagctgatgtggaaggcagagaaggattctttcttccagccctggatctccttctccataaagaacaaagaggtgtccgtacaaaagtccaccaaagacctcaagctccagctgaaggaaacgctcccactcaccctcaagataccccaggtctcgcttcagtttgctggttctggcaacctgactctgactctggacaaagggacactgcatcaggaagtgaacctggtggtgatgaaagtggctcagctcaacaatactttgacctgtgaggtgatgggacctacctctcccaagatgagactgaccctgaagcaggagaaccaggaggccagggtctctgaggagcagaaagtagttcaagtggtggcccctgagacagggctgtggcagtgtctactgagtgaaggtgataaggtcaagatggactccaggatccaggttttatccagaggggtgaaccagacagtgttcctggcttgcgtgctgggt**GTGtccttcCTC**tttctgggtttccttgggctctgcatcctctgc**TCTgtcaggTCC**cggcaccaacagcgccaggcagcacgaatgtctcagatcaagaggctcctcagtgagaagaagacctgccagtgcccccaccggatgcagaagagccataatctcatctaatgaagatcttgagtcgacaagcttgctag

**TPC**:

acggaattccgctcgagcgccaccatggtgcgagccatctctcttaggcgcttgctgctgctgctgctgcagctgtcacaactcctagctgtcactcaagggaagacgctggtgctggggaaggaaggggaatcagcagaactgccctgcgagagttcccagaagaagatcacagtcttcacctggaagttctctgaccagaggaagattctggggcagcatggcaaaggtgtattaattagaggaggttcgccttcgcagtttgatcgttttgattccaaaaaaggggcatgggagaaaggatcgtttcctctcatcatcaataaacttaagatggaagactctcagacttatatctgtgagctggagaacaggaaagaggaggtggagttgtgggtgttcaaagtgaccttcagtccgggtaccagcctgttgcaagggcagagcctgaccctgaccttggatagcaactctaaggtctctaaccccttgacagagtgcaaacacaaaaagggtaaagttgtcagtggttccaaagttctctccatgtccaacctaagggttcaggacagcgacttctggaactgcaccgtgaccctggaccagaaaaagaactggttcggcatgacactctcagtgctgggttttcagagcacagctatcacggcctataagagtgagggagagtcagcggagttctccttcccactcaactttgcagaggaaaacgggtggggagagctgatgtggaaggcagagaaggattctttcttccagccctggatctccttctccataaagaacaaagaggtgtccgtacaaaagtccaccaaagacctcaagctccagctgaaggaaacgctcccactcaccctcaagataccccaggtctcgcttcagtttgctggttctggcaacctgactctgactctggacaaagggacactgcatcaggaagtgaacctggtggtgatgaaagtggctcagctcaacaatactttgacctgtgaggtgatgggacctacctctcccaagatgagactgaccctgaagcaggagaaccaggaggccagggtctctgaggagcagaaagtagttcaagtggtggcccctgagacagggctgtggcagtgtctactgagtgaaggtgataaggtcaagatggactccaggatccaggttttatccagaggggtgaaccagacagtgttcctggcttgcgtgctgggt**GTGtccttcCTC**tttctgggtttccttgggctctgcatcctctgc**TCTgtcaggTCC**cggcaccaacagcgccaggcagcacgaatgtctcagatcaagaggctcctcagtgagaagaagacc**TCCcagTCC**ccccaccggatgcagaagagccataatctcatctaatgaagatcttgagtcgacaagcttgctag

**pSS**:

acggaattccgctcgagcgccaccatggtgcgagccatctctcttaggcgcttgctgctgctgctgctgcagctgtcacaactcctagctgtcactcaagggaagacgctggtgctggggaaggaaggggaatcagcagaactgccctgcgagagttcccagaagaagatcacagtcttcacctggaagttctctgaccagaggaagattctggggcagcatggcaaaggtgtattaattagaggaggttcgccttcgcagtttgatcgttttgattccaaaaaaggggcatgggagaaaggatcgtttcctctcatcatcaataaacttaagatggaagactctcagacttatatctgtgagctggagaacaggaaagaggaggtggagttgtgggtgttcaaagtgaccttcagtccgggtaccagcctgttgcaagggcagagcctgaccctgaccttggatagcaactctaaggtctctaaccccttgacagagtgcaaacacaaaaagggtaaagttgtcagtggttccaaagttctctccatgtccaacctaagggttcaggacagcgacttctggaactgcaccgtgaccctggaccagaaaaagaactggttcggcatgacactctcagtgctgggttttcagagcacagctatcacggcctataagagtgagggagagtcagcggagttctccttcccactcaactttgcagaggaaaacgggtggggagagctgatgtggaaggcagagaaggattctttcttccagccctggatctccttctccataaagaacaaagaggtgtccgtacaaaagtccaccaaagacctcaagctccagctgaaggaaacgctcccactcaccctcaagataccccaggtctcgcttcagtttgctggttctggcaacctgactctgactctggacaaagggacactgcatcaggaagtgaacctggtggtgatgaaagtggctcagctcaacaatactttgacctgtgaggtgatgggacctacctctcccaagatgagactgaccctgaagcaggagaaccaggaggccagggtctctgaggagcagaaagtagttcaagtggtggcccctgagacagggctgtggcagtgtctactgagtgaaggtgataaggtcaagatggactccaggatccaggttttatccagaggggtgaaccagacagtgttcctggcttgcgtgctgggtggctccttcggctttctgggtttccttgggctctgcatcctctgctgtgtcaggtgccggcaccaacagcgccaggcagcacgaatg**GACcagatcaagaggctcctcGAC**gagaagaagacctgccagtgcccccaccggatgcagaagagccataatctcatctaatgaagatcttgagtcgacaagcttgctag

**LL+pSS**:

acggaattccgctcgagcgccaccatggtgcgagccatctctcttaggcgcttgctgctgctgctgctgcagctgtcacaactcctagctgtcactcaagggaagacgctggtgctggggaaggaaggggaatcagcagaactgccctgcgagagttcccagaagaagatcacagtcttcacctggaagttctctgaccagaggaagattctggggcagcatggcaaaggtgtattaattagaggaggttcgccttcgcagtttgatcgttttgattccaaaaaaggggcatgggagaaaggatcgtttcctctcatcatcaataaacttaagatggaagactctcagacttatatctgtgagctggagaacaggaaagaggaggtggagttgtgggtgttcaaagtgaccttcagtccgggtaccagcctgttgcaagggcagagcctgaccctgaccttggatagcaactctaaggtctctaaccccttgacagagtgcaaacacaaaaagggtaaagttgtcagtggttccaaagttctctccatgtccaacctaagggttcaggacagcgacttctggaactgcaccgtgaccctggaccagaaaaagaactggttcggcatgacactctcagtgctgggttttcagagcacagctatcacggcctataagagtgagggagagtcagcggagttctccttcccactcaactttgcagaggaaaacgggtggggagagctgatgtggaaggcagagaaggattctttcttccagccctggatctccttctccataaagaacaaagaggtgtccgtacaaaagtccaccaaagacctcaagctccagctgaaggaaacgctcccactcaccctcaagataccccaggtctcgcttcagtttgctggttctggcaacctgactctgactctggacaaagggacactgcatcaggaagtgaacctggtggtgatgaaagtggctcagctcaacaatactttgacctgtgaggtgatgggacctacctctcccaagatgagactgaccctgaagcaggagaaccaggaggccagggtctctgaggagcagaaagtagttcaagtggtggcccctgagacagggctgtggcagtgtctactgagtgaaggtgataaggtcaagatggactccaggatccaggttttatccagaggggtgaaccagacagtgttcctggcttgcgtgctgggtggctccttcggctttctgggtttccttgggctctgcatcctctgctgtgtcaggtgccggcaccaacagcgccaggcagcacgaatg**GACcagatcaagagggctgcaGAC**gagaagaagacctgccagtgcccccaccggatgcagaagagccataatctcatctgataaagatcttgagtcgacaagcttgctag

**CD4-T1 (note – for construct requests this is called CD4Tr2 in Kuhns Lab library to distinguish from previously published truncated constructs):**

acggaattccgctcgagcgccaccatggtgcgagccatctctcttaggcgcttgctgctgctgctgctgcagctgtcacaactcctagctgtcactcaagggaagacgctggtgctggggaaggaaggggaatcagcagaactgccctgcgagagttcccagaagaagatcacagtcttcacctggaagttctctgaccagaggaagattctggggcagcatggcaaaggtgtattaattagaggaggttcgccttcgcagtttgatcgttttgattccaaaaaaggggcatgggagaaaggatcgtttcctctcatcatcaataaacttaagatggaagactctcagacttatatctgtgagctggagaacaggaaagaggaggtggagttgtgggtgttcaaagtgaccttcagtccgggtaccagcctgttgcaagggcagagcctgaccctgaccttggatagcaactctaaggtctctaaccccttgacagagtgcaaacacaaaaagggtaaagttgtcagtggttccaaagttctctccatgtccaacctaagggttcaggacagcgacttctggaactgcaccgtgaccctggaccagaaaaagaactggttcggcatgacactctcagtgctgggttttcagagcacagctatcacggcctataagagtgagggagagtcagcggagttctccttcccactcaactttgcagaggaaaacgggtggggagagctgatgtggaaggcagagaaggattctttcttccagccctggatctccttctccataaagaacaaagaggtgtccgtacaaaagtccaccaaagacctcaagctccagctgaaggaaacgctcccactcaccctcaagataccccaggtctcgcttcagtttgctggttctggcaacctgactctgactctggacaaagggacactgcatcaggaagtgaacctggtggtgatgaaagtggctcagctcaacaatactttgacctgtgaggtgatgggacctacctctcccaagatgagactgaccctgaagcaggagaaccaggaggccagggtctctgaggagcagaaagtagttcaagtggtggcccctgagacagggctgtggcagtgtctactgagtgaaggtgataaggtcaagatggactccaggatccaggttttatccagaggggtgaaccagacagtgttcctggcttgcgtgctgggtggctccttcggctttctgggtttccttgggctctgcatcctctgctgtgtcaggtgccggtgataaagatcttgagtcgacaagcttgctag

Gene blocks were purchased from Twist Biosciences that encode the required mutations in each of the following CD4 genes H, HC, LL, SS, LL+TP. The gene segments were cloned into pUC18 containing CD4 WT whereby the gene segments flanked by BamHI and HindIII were replaced with the mutant geneblocks. The genes were sequence verified in pUC 18, subcloned into pP2 puromycin-resistance MSCV vector (MCS-IRES-puro) via 5’XhoI and 3’BglII, midi-prepped, and sequenced. The sequences for these constructs are listed below. Mutated codons are shown in bold uppercase letters, while the motif under interrogation is shown in bold and underlined (any non-mutated codons within the motif are bold lowercase letters and underlined):

**H**:

acggaattccgctcgagcgccaccatggtgcgagccatctctcttaggcgcttgctgctgctgctgctgcagctgtcacaactcctagctgtcactcaagggaagacgctggtgctggggaaggaaggggaatcagcagaactgccctgcgagagttcccagaagaagatcacagtcttcacctggaagttctctgaccagaggaagattctggggcagcatggcaaaggtgtattaattagaggaggttcgccttcgcagtttgatcgttttgattccaaaaaaggggcatgggagaaaggatcgtttcctctcatcatcaataaacttaagatggaagactctcagacttatatctgtgagctggagaacaggaaagaggaggtggagttgtgggtgttcaaagtgaccttcagtccgggtaccagcctgttgcaagggcagagcctgaccctgaccttggatagcaactctaaggtctctaaccccttgacagagtgcaaacacaaaaagggtaaagttgtcagtggttccaaagttctctccatgtccaacctaagggttcaggacagcgacttctggaactgcaccgtgaccctggaccagaaaaagaactggttcggcatgacactctcagtgctgggttttcagagcacagctatcacggcctataagagtgagggagagtcagcggagttctccttcccactcaactttgcagaggaaaacgggtggggagagctgatgtggaaggcagagaaggattctttcttccagccctggatctccttctccataaagaacaaagaggtgtccgtacaaaagtccaccaaagacctcaagctccagctgaaggaaacgctcccactcaccctcaagataccccaggtctcgcttcagtttgctggttctggcaacctgactctgactctggacaaagggacactgcatcaggaagtgaacctggtggtgatgaaagtggctcagctcaacaatactttgacctgtgaggtgatgggacctacctctcccaagatgagactgaccctgaagcaggagaaccaggaggccagggtctctgaggagcagaaagtagttcaagtggtggcccctgagacagggctgtggcagtgtctactgagtgaaggtgataaggtcaagatggactccaggatccaggttttatccagaggggtgaaccagacagtgttcctggcttgcgtgctgggtggctccttcggctttctgggtttccttgggctctgcatcctctgctgtgtcaggtgccggcaccaacagcgccaggcagca**AACGGCCCTGGTGGCAATCCTGGTGGCAACGCAGGCGGT**acctgccagtgcccccaccggatgcagaagagccataatctcatctgataaagatcttgagtcgacaagcttgctag

**HC**:

acggaattccgctcgagcgccaccatggtgcgagccatctctcttaggcgcttgctgctgctgctgctgcagctgtcacaactcctagctgtcactcaagggaagacgctggtgctggggaaggaaggggaatcagcagaactgccctgcgagagttcccagaagaagatcacagtcttcacctggaagttctctgaccagaggaagattctggggcagcatggcaaaggtgtattaattagaggaggttcgccttcgcagtttgatcgttttgattccaaaaaaggggcatgggagaaaggatcgtttcctctcatcatcaataaacttaagatggaagactctcagacttatatctgtgagctggagaacaggaaagaggaggtggagttgtgggtgttcaaagtgaccttcagtccgggtaccagcctgttgcaagggcagagcctgaccctgaccttggatagcaactctaaggtctctaaccccttgacagagtgcaaacacaaaaagggtaaagttgtcagtggttccaaagttctctccatgtccaacctaagggttcaggacagcgacttctggaactgcaccgtgaccctggaccagaaaaagaactggttcggcatgacactctcagtgctgggttttcagagcacagctatcacggcctataagagtgagggagagtcagcggagttctccttcccactcaactttgcagaggaaaacgggtggggagagctgatgtggaaggcagagaaggattctttcttccagccctggatctccttctccataaagaacaaagaggtgtccgtacaaaagtccaccaaagacctcaagctccagctgaaggaaacgctcccactcaccctcaagataccccaggtctcgcttcagtttgctggttctggcaacctgactctgactctggacaaagggacactgcatcaggaagtgaacctggtggtgatgaaagtggctcagctcaacaatactttgacctgtgaggtgatgggacctacctctcccaagatgagactgaccctgaagcaggagaaccaggaggccagggtctctgaggagcagaaagtagttcaagtggtggcccctgagacagggctgtggcagtgtctactgagtgaaggtgataaggtcaagatggactccaggatccaggttttatccagaggggtgaaccagacagtgttcctggcttgcgtgctgggtggctccttcggctttctgggtttccttgggctctgcatcctctgctgtgtcaggtgccggcaccaacagcgccaggcagca**AACGGCCCTGGTGGCAATCCTGGTGGCAACGCAGGCGGT**acc**TCCcagTCC**ccccaccggatgcagaagagccataatctcatctgataaagatcttgagtcgacaagcttgctag

**LL**:

acggaattccgctcgagcgccaccatggtgcgagccatctctcttaggcgcttgctgctgctgctgctgcagctgtcacaactcctagctgtcactcaagggaagacgctggtgctggggaaggaaggggaatcagcagaactgccctgcgagagttcccagaagaagatcacagtcttcacctggaagttctctgaccagaggaagattctggggcagcatggcaaaggtgtattaattagaggaggttcgccttcgcagtttgatcgttttgattccaaaaaaggggcatgggagaaaggatcgtttcctctcatcatcaataaacttaagatggaagactctcagacttatatctgtgagctggagaacaggaaagaggaggtggagttgtgggtgttcaaagtgaccttcagtccgggtaccagcctgttgcaagggcagagcctgaccctgaccttggatagcaactctaaggtctctaaccccttgacagagtgcaaacacaaaaagggtaaagttgtcagtggttccaaagttctctccatgtccaacctaagggttcaggacagcgacttctggaactgcaccgtgaccctggaccagaaaaagaactggttcggcatgacactctcagtgctgggttttcagagcacagctatcacggcctataagagtgagggagagtcagcggagttctccttcccactcaactttgcagaggaaaacgggtggggagagctgatgtggaaggcagagaaggattctttcttccagccctggatctccttctccataaagaacaaagaggtgtccgtacaaaagtccaccaaagacctcaagctccagctgaaggaaacgctcccactcaccctcaagataccccaggtctcgcttcagtttgctggttctggcaacctgactctgactctggacaaagggacactgcatcaggaagtgaacctggtggtgatgaaagtggctcagctcaacaatactttgacctgtgaggtgatgggacctacctctcccaagatgagactgaccctgaagcaggagaaccaggaggccagggtctctgaggagcagaaagtagttcaagtggtggcccctgagacagggctgtggcagtgtctactgagtgaaggtgataaggtcaagatggactccaggatccaggttttatccagaggggtgaaccagacagtgttcctggcttgcgtgctgggtggctccttcggctttctgggtttccttgggctctgcatcctctgctgtgtcaggtgccggcaccaacagcgccaggcagcacgaatgtctcag**atcaagaggGCTGCA**agtgagaagaagacctgccagtgcccccaccggatgcagaagagccataatctcatctgataaagatcttgagtcgacaagcttgctag

**SS**:

acggaattccgctcgagcgccaccatggtgcgagccatctctcttaggcgcttgctgctgctgctgctgcagctgtcacaactcctagctgtcactcaagggaagacgctggtgctggggaaggaaggggaatcagcagaactgccctgcgagagttcccagaagaagatcacagtcttcacctggaagttctctgaccagaggaagattctggggcagcatggcaaaggtgtattaattagaggaggttcgccttcgcagtttgatcgttttgattccaaaaaaggggcatgggagaaaggatcgtttcctctcatcatcaataaacttaagatggaagactctcagacttatatctgtgagctggagaacaggaaagaggaggtggagttgtgggtgttcaaagtgaccttcagtccgggtaccagcctgttgcaagggcagagcctgaccctgaccttggatagcaactctaaggtctctaaccccttgacagagtgcaaacacaaaaagggtaaagttgtcagtggttccaaagttctctccatgtccaacctaagggttcaggacagcgacttctggaactgcaccgtgaccctggaccagaaaaagaactggttcggcatgacactctcagtgctgggttttcagagcacagctatcacggcctataagagtgagggagagtcagcggagttctccttcccactcaactttgcagaggaaaacgggtggggagagctgatgtggaaggcagagaaggattctttcttccagccctggatctccttctccataaagaacaaagaggtgtccgtacaaaagtccaccaaagacctcaagctccagctgaaggaaacgctcccactcaccctcaagataccccaggtctcgcttcagtttgctggttctggcaacctgactctgactctggacaaagggacactgcatcaggaagtgaacctggtggtgatgaaagtggctcagctcaacaatactttgacctgtgaggtgatgggacctacctctcccaagatgagactgaccctgaagcaggagaaccaggaggccagggtctctgaggagcagaaagtagttcaagtggtggcccctgagacagggctgtggcagtgtctactgagtgaaggtgataaggtcaagatggactccaggatccaggttttatccagaggggtgaaccagacagtgttcctggcttgcgtgctgggtggctccttcggctttctgggtttccttgggctctgcatcctctgctgtgtcaggtgccggcaccaacagcgccaggcagcacgaatg**GCTcagatcaagaggctcctcGCA**gagaagaagacctgccagtgcccccaccggatgcagaagagccataatctcatctgataaagatcttgagtcgacaagcttgctag

**LL+TP**:

acggaattccgctcgagcgccaccatggtgcgagccatctctcttaggcgcttgctgctgctgctgctgcagctgtcacaactcctagctgtcactcaagggaagacgctggtgctggggaaggaaggggaatcagcagaactgccctgcgagagttcccagaagaagatcacagtcttcacctggaagttctctgaccagaggaagattctggggcagcatggcaaaggtgtattaattagaggaggttcgccttcgcagtttgatcgttttgattccaaaaaaggggcatgggagaaaggatcgtttcctctcatcatcaataaacttaagatggaagactctcagacttatatctgtgagctggagaacaggaaagaggaggtggagttgtgggtgttcaaagtgaccttcagtccgggtaccagcctgttgcaagggcagagcctgaccctgaccttggatagcaactctaaggtctctaaccccttgacagagtgcaaacacaaaaagggtaaagttgtcagtggttccaaagttctctccatgtccaacctaagggttcaggacagcgacttctggaactgcaccgtgaccctggaccagaaaaagaactggttcggcatgacactctcagtgctgggttttcagagcacagctatcacggcctataagagtgagggagagtcagcggagttctccttcccactcaactttgcagaggaaaacgggtggggagagctgatgtggaaggcagagaaggattctttcttccagccctggatctccttctccataaagaacaaagaggtgtccgtacaaaagtccaccaaagacctcaagctccagctgaaggaaacgctcccactcaccctcaagataccccaggtctcgcttcagtttgctggttctggcaacctgactctgactctggacaaagggacactgcatcaggaagtgaacctggtggtgatgaaagtggctcagctcaacaatactttgacctgtgaggtgatgggacctacctctcccaagatgagactgaccctgaagcaggagaaccaggaggccagggtctctgaggagcagaaagtagttcaagtggtggcccctgagacagggctgtggcagtgtctactgagtgaaggtgataaggtcaagatggactccaggatccaggttttatccagaggggtgaaccagacagtgttcctggcttgcgtgctgggt**GTGtccttcCTC**tttctgggtttccttgggctctgcatcctctgc**TCTgtcaggTCC**cggcaccaacagcgccaggcagcacgaatgtctcag**atcaagaggGCTGCA**agtgagaagaagacctgccagtgcccccaccggatgcagaagagccataatctcatctaatgaagatcttgagtcgacaagcttgctag

The genes of LL+ELXE and LL+Δbind were made by cloning the in frame extracellular domain of CD4 that encodes PLXF to ELXF (Glassman et al., 2018) or Δbind extracellular domain (Parrish et al., 2015) flanked by XhoI and BamHI into the CD4LL gene. The genes were sequence verified in pUC 18, subcloned into pP2 puromycin-resistance MSCV vector (MCS-IRES-puro) via 5’XhoI and 3’BglII, midi-prepped, and sequenced. The sequences for these constructs are as follows with mutated codons shown in bold uppercase letters, while the motif under interrogation is shown in bold and underlined (any non-mutated codons within the motif are bold lowercase letters and underlined):

**LL+ELXE**:

acggaattccgctcgagcgccaccatggtgcgagccatctctcttaggcgcttgctgctgctgctgctgcagctgtcacaactcctagctgtcactcaagggaagacgctggtgctggggaaggaaggggaatcagcagaactgccctgcgagagttcccagaagaagatcacagtcttcacctggaagttctctgaccagaggaagattctggggcagcatggcaaaggtgtattaattagaggaggttcgccttcgcagtttgatcgttttgattccaaaaaaggggcatgggagaaaggatcgtttcctctcatcatcaataaacttaagatggaagactctcagacttatatctgtgagctggagaacaggaaagaggaggtggagttgtgggtgttcaaagtgaccttcagtccgggtaccagcctgttgcaagggcagagcctgaccctgaccttggatagcaactctaaggtctctaaccccttgacagagtgcaaacacaaaaagggtaaagttgtcagtggttccaaagttctctccatgtccaacctaagggttcaggacagcgacttctggaactgcaccgtgaccctggaccagaaaaagaactggttcggcatgacactctcagtgctgggttttcagagcacagctatcacggcctataagagtgagggagagtcagcggagttctccttc**GAActcaacGAG**gcagaggaaaacgggtggggagagctgatgtggaaggcagagaaggattctttcttccagccctggatctccttctccataaagaacaaagaggtgtccgtacaaaagtccaccaaagacctcaagctccagctgaaggaaacgctcccactcaccctcaagataccccaggtctcgcttcagtttgctggttctggcaacctgactctgactctggacaaagggacactgcatcaggaagtgaacctggtggtgatgaaagtggctcagctcaacaatactttgacctgtgaggtgatgggacctacctctcccaagatgagactgaccctgaagcaggagaaccaggaggccagggtctctgaggagcagaaagtagttcaagtggtggcccctgagacagggctgtggcagtgtctactgagtgaaggtgataaggtcaagatggactccaggatccaggttttatccagaggggtgaaccagacagtgttcctggcttgcgtgctgggtggctccttcggctttctgggtttccttgggctctgcatcctctgctgtgtcaggtgccggcaccaacagcgccaggcagcacgaatgtctcag**atcaagaggGCTGCA**agtgagaagaagacctgccagtgcccccaccggatgcagaagagccataatctcatctgataaagatcttgagtcgacaagcttgctag

**LL+Δbind**:

acggaattccgctcgagcgccaccatggtgcgagccatctctcttaggcgcttgctgctgctgctgctgcagctgtcacaactcctagctgtcactcaagggaagacgctggtgctggggaaggaaggggaatcagcagaactgccctgcgagagttcccagaagaagatcacagtcttcacctggaagttctctgaccagaggaagattctggggcagcatggc**GATggtGATTCAGATAGC**ggaggttcgccttcgcagtttgatcgttttgattccaaaaaaggggcatgggagaaaggatcgtttcctctcatcatcaataaacttaagatggaagactctcagacttatatctgtgagctggagaacaggaaagaggaggtggagttgtgggtgttcaaagtgaccttcagtccgggtaccagcctgttgcaagggcagagcctgaccctgaccttggatagcaactctaaggtctctaaccccttgacagagtgcaaacacaaaaagggtaaagttgtcagtggttccaaagttctctccatgtccaacctaagggttcaggacagcgacttctggaactgcaccgtgaccctggaccagaaaaagaactggttcggcatgacactctcagtgctgggttttcagagcacagctatcacggcctataagagtgagggagagtcagcggagttctccttcccactcaactttgcagaggaaaacgggtggggagagctgatgtggaaggcagagaaggattctttcttccagccctggatctccttctccataaagaacaaagaggtgtccgtacaaaagtccaccaaagacctcaagctccagctgaaggaaacgctcccactcaccctcaagataccccaggtctcgcttcagtttgctggttctggcaacctgactctgactctggacaaagggacactgcatcaggaagtgaacctggtggtgatgaaagtggctcagctcaacaatactttgacctgtgaggtgatgggacctacctctcccaagatgagactgaccctgaagcaggagaaccaggaggccagggtctctgaggagcagaaagtagttcaagtggtggcccctgagacagggctgtggcagtgtctactgagtgaaggtgataaggtcaagatggactccaggatccaggttttatccagaggggtgaaccagacagtgttcctggcttgcgtgctgggtggctccttcggctttctgggtttccttgggctctgcatcctctgctgtgtcaggtgccggcaccaacagcgccaggcagcacgaatgtctcag**atcaagaggGCTGCA**agtgagaagaagacctgccagtgcccccaccggatgcagaagagccataatctcatctgataaagatcttgagtcgacaagcttgctag
